# Supplementary material for: Are carbon emissions trading and green financial instruments synergistic? -Comprehensive quantitative research based on content analysis
Source: PLoS One. 2024 Mar 7;19(3):e0298601. doi: 10.1371/journal.pone.0298601 (PMC10919601; doi:10.1371/journal.pone.0298601)
Supplement: S1 Data — (ZIP) [file pone.0298601.s001.zip › Data/地级市Z_F工作报告.zip!/╔╜╬≈/╠1⁄2╘¡╩╨/└┤╫╘╣1⁄2╓┌║┼í╛┬φ┐╦╩2╛▌═°í┐_2012.docx]

各位代表：

现在，我代表市政府向大会作报告，请予审议，并请政协委员和列席人员提出意见。

一、本届政府工作回顾

本届政府自2007年5月市第十二届人民代表大会第一次会议选举产生以来，任期届满。

五年来，在省委、省政府和市委的正确领导下，在市人大和市政协的有力监督支持下，市政府深入贯彻落实科学发展观，团结带领全市人民迎难而上，奋力拼搏，创新发展模式，推进绿色转型，努力建设一流的省会城市和具有国际影响力的区域性现代化大都市，国民经济和社会发展迈上新台阶，完成了“十一五”规划的目标任务，实现了“十二五”规划的良好开局。

五年来，全市地区生产总值由1042亿元增加到2080亿元，年均增长9.7%；财政总收入由192.2亿元增加到393亿元，年均增长15.4%；一般预算收入由75.3亿元增加到174.7亿元，年均增长18.3%；规模以上工业增加值由335.7亿元增加到687.2亿元，年均增长9%；固定资产投资累计达到4002亿元，年均增长15.4%；社会消费品零售总额由436.5亿元增加到973.3亿元，年均增长17.4%；外贸进出口总额由41.1亿美元增加到85.3亿美元，年均增长15.7%；城镇居民人均可支配收入由11741元增加到20149元，年均增长11.4%；农民人均纯收入由4917元增加到8888元，年均增长12.6%。

五年来，我们牢牢把握科学发展的主动权，积极应对国际金融危机冲击，保增长、保民生、保稳定和促转型工作取得实效，综合经济实力显著增强。深入推进产业结构调整，煤炭资源整合、煤矿兼并重组取得重大成果，一流新兴产业基地建设迈出新步伐；现代服务业快速发展，消费市场繁荣活跃；农业产业化加速推进，农民收入不断提高，农村“五个全覆盖”全部实现，新农村建设扎实有效。城市基础建设力度加大，一批重大标志性项目陆续竣工，长风商务区初具规模，城市面貌明显改变；创新城乡管理体制机制，大力开展城乡清洁工程，城市综合承载能力不断提升。环境保护与生态建设有效推进，节能减排任务超额完成，人居环境得到改善，资源节约和环境友好型社会建设取得新进展。改革开放深入推进，国企改革攻坚任务基本完成，成功承办“中博会”等重大活动，城市形象得到提升；创新能力不断提高，深入开展政务环境“对标”活动，发展环境持续优化。社会事业稳步发展，基本公共服务体系日趋健全，民生得到较好保障和改善，改革发展成果惠及更多群众。政府与企业的两个主体责任得到有效落实，安全生产形势持续稳定好转；加强与创新社会管理取得实效，社会保持和谐稳定。

五年来，我市成功进入国家循环经济标准化试点市、国家创新型城市试点和国家知识产权示范市、全国数字城市示范市、国家第一批可再生能源建筑应用示范城市、国家历史文化名城行列。先后荣获国家园林城市、全国文化体制改革先进地区、全国未成年人思想道德建设工作先进城市、全国人民防空先进城市等荣誉称号，城市的影响力进一步增强。

各位代表，2007—2010年工作已逐年向大会报告，在对本届政府五年工作简要回顾的基础上，现重点就2011年政府工作向大会报告。

2011年，市政府认真贯彻落实建设一流省会城市的战略部署，全力抓好“十二五”规划开局工作，经济社会发展取得了新的成绩。全市地区生产总值增长9.9%；规模以上工业增加值增长12.2%；固定资产投资增长25.1%，达到1024.1亿元；社会消费品零售总额增长17.9%；地区外贸进出口总额增长7.9%；财政总收入增长22.1%，一般预算收入增长26.2%；城镇居民人均可支配收入增长16.8%；农民人均纯收入增长16.8%。

（一）一流新兴产业基地建设加快推进，新兴产业加快发展，经济发展方式加快转变。

投资结构更加优化。狠抓项目建设，固定资产投资比“十一五”年均完成量高出328.43亿元，投资增速比“十一五”平均增速高出9.2个百分点。第三产业完成投资720.29亿元，是“十一五”年均投资额的1.9倍，占总投资比重70.3%。工业投资增速达到30.2%，重回快速增长的轨道，其中以装备制造业、新材料等为代表的新兴产业投资增长28.6%，高出“十一五”年均增速20.3个百分点；新兴产业投资占工业投资的比重由2006年的29.4%提高到46.7%；内涵型工业项目投资增长44.5%，高出外延型投资18.7个百分点。

工业转型步伐加快。新兴产业发展速度加快，增加值增速达到27.7%，高于煤焦铁化电等传统产业19.3个百分点；占工业比重18.5%，对工业经济增长的贡献率达到40.9%，比上年提高9.2个百分点。工业经济效益综合指数达到196.17，比上年提高10.59点。高新区4家动漫企业进入国家级行列。经济区获批“国家新材料高新技术产业化基地”、“国家铝镁合金外贸转型升级示范基地”。民营区工业新区加快建设，引进晋西装备产业园等一批新兴项目。不锈钢园区一批优势项目相继落户。清徐经济开发区阳煤集团化工新材料园奠基。

第三产业快速发展。服务业增加值实现1097.1亿元，增长8.7%。成功举办第二届农博会、太原国际汽车展等会展，获得“2011年度中国十佳品牌会展城市”称号。民营区成功创建国家级物流服务标准化试点并通过验收；盛唐物流冷链加工配送等一批物流项目投产达效。国家家政服务体系建设项目试点工作进展顺利，141户家政企业纳入服务网络。全市再生资源回收体系基本建立。实现旅游总收入276.7亿元，增长20.1%。

都市农业稳步推进。确定娄烦县马铃薯、小店区奶牛和清徐县蔬菜3个“一县一业”特色基地县和64个“一村一品”专业示范村。3个国家级农业标准化示范区均顺利通过验收。全市无公害农产品基地达到46万亩。建设节水农业5.6万亩。改扩建、新建规模健康养殖示范场61个。大力发展食醋、肉制品、干鲜果、蔬菜和优质粮油五大加工业，农产品加工销售收入91.9亿元，增长31.2%。新发展农民专业合作社338个。实施扶贫项目150个，脱贫1.9万人。

民营经济加快发展。制定出台《关于大力推进民营经济转型跨越发展的意见》，加大政策扶持力度，破解融资难题，为民营经济融资280亿元，其中通过担保公司和小贷公司融资108亿元。现代物流、技术服务、设施农业等成为民营经济新的增长点。国家工信部微小企业创业园落户太原。百圆裤业成为我市第一家在深交所成功上市的民营企业。

（二）一流自主创新基地建设加快推进，科技创新能力增强，支撑作用不断提升。

科技创新能力增强。全社会研究与开发经费投入59.8亿元，增长16.8%。全市专利申请量6525件、授权量3012件，均增长30%。经省鉴定的科技成果156项，增长20%，其中68%达到国内领先水平。引进5名国家“千人计划”人才，建设“云计算应用平台及实验室”等15个企业工程实验室（中试车间）和2个国际科技合作基地。269个项目列入国家各类科技计划。荣膺“全国科技进步先进市”称号。

科技支撑作用提升。实施战略性新兴产业科技项目194项。高速铁路车辆轮轴用钢工艺技术开发等26个重大自主创新项目、液态金属芯片散热器等30个高新技术产业化项目取得突破性进展。开展“中小企业自主创新能力建设年”活动，150余家中小企业实施信息化改造。国家级企业技术中心达到6户，通泽重工技术中心成为我省首家民营企业国家级技术中心。信联集团、中绿环保公司成为国家创新型企业，火炬来特公司成为首批国家现代服务业创新发展示范企业。高新技术产业增加值增长18.7%。

科技成果转化加快。搭建科技成果转化平台。新培育4家产学研战略联盟和3个产业集群技术联盟，新发展技术服务机构40家。大力推广应用民生科技成果。科技成果转化率达到55.2%，比上年提高4.9个百分点。

（三）一流现代宜居城市建设力度加大，城乡面貌加快改善，承载能力不断提高。

城市规划作用得到强化。土地利用总体规划与城市总体规划同步升级，实现与产业规划、环境保护规划“四规合一”。编制完成南部区域发展战略规划、太原都市区规划和重点地段城市设计，城市综合交通规划进一步完善，城中村改造、保障性住房等民生工程规划编制全面展开。实现中心城区589平方公里控规全覆盖。

城乡基础建设加快推进。新增建设用地3.1万亩，同比增长21%；实现国土收益90亿元。开工建设各类城建重点工程项目336项，长风商务区等一批新地标相继建成。滨河西路南延19.5公里竣工通车。完成汾河治理美化南延工程，形成纵贯市区长达20.5公里的绿色长廊。城中村改造有序推进，启动拆迁24个村。城镇化建设扎实推进，徐沟镇和马兰镇列入全省“百镇建设”示范镇。

城乡管理水平不断提升。深入推进城乡清洁工程，全面开展达标活动，围绕“十纵、十横、十片区”等重点区域和重点地段，集中开展街景立面、市容环卫等10多项整治，市容环境明显改观。大力推进“净化太原”、“气化太原”和“供热全覆盖”建设，集中供热扩网1323万平方米，比上年增长21%，集中供热普及率达87%。

节能减排与环境保护效果显著。加大污染减排力度，实施41项重点减排工程，关停22家工业企业49台（套）生产设施。太化合成氨全面停产，狮头水泥等一批重点污染企业搬迁工作全面启动。万元地区生产总值能耗下降3.52%，万元工业增加值用水量下降5%。六项污染物减排指标均完成省下达的任务。市区环境空气综合污染指数下降3.5%；二级以上天数达到308天，比上年增加4天。

生态建设成效明显。完成造林49.2万亩，相当于“十一五”后三年的总和。巩固发展“国家园林城市”成果，实施长风商务区绿化等重点工程31项，新增绿化覆盖面积363公顷，新增绿化面积354公顷，新增公园绿地面积150公顷。治理水土流失面积23.5万亩。市区水环境功能区水质达标率达到62.5%，集中式饮用水源地水质达标率保持100%。孟封镇、东高白村分别被确定为国家级生态乡镇、国家级生态村。

（四）改革开放不断深化，政务环境持续优化，发展的动力与活力不断增强。

各项改革不断深化。编制完成《太原市转型综改试验行动方案》，首批转型综改标杆项目加快推进。清徐、古交列入全省扩权强县试点县。创新生态绿化机制，引进14家企业建设西山城郊森林公园。加快城市建设投融资体制改革，完成四个融资平台的清理规范工作。农村土地经营权流转面积27万亩。集体林权制度改革确权面积121万亩。历时三年的公安警务运行机制改革圆满完成。城乡建设用地增减挂钩试点初步推开。强化财税管理，优化调整支出结构，民生支出194.8亿元，增长35%，占一般预算支出比例达到81.4%。

对外交流与合作不断扩大。加大招商引资力度，积极组团参加各类大型经贸合作洽谈会，支持外贸企业拓展海外市场。新批设立外商投资企业20户，实际利用外资6.8亿美元，增长16.1%。实际利用境内资金376.3亿元人民币，增长61%。

效能政府建设取得新突破。以流程再造、绩效考核和行政文化建设为重点，开展政务环境“对标”活动，减少审批事项215项，减幅56.1%。对行政审批流程进行体系化和信息化再造，完成新的固定资产投资项目联合审批流程图，审批时间由320个工作日缩短为45个工作日。

（五）民生和社会事业发展全面加快，社会管理加强与创新进一步强化，和谐社会建设迈出新步伐。

民生改善力度加大。新开工建设保障性住房45870套，是省定任务的2.67倍。新建、改扩建“百校兴学”工程项目学校202所、公办幼儿园31所，“百校兴学”工程荣获全国教育改革创新特别奖。在48个乡镇卫生院建立“三位一体”远程诊断系统，在50个社区卫生服务机构设置自助式检测健康小屋。新建设施蔬菜1.54万亩，建成40个标准化菜市场。新购置600台公交车，新开通6条公交线路。改善和提高10万农村人口的饮水标准。为2400名贫困残疾人提供康复救助，为118个社区配发残疾人康复训练器材，为260户农村贫困残疾人家庭实施危房改造。为1万名生活困难的空巢老人配发“爱心一键通”。新建公厕100座。为540个社区文化室配备活动器材。加强社会救助体系建设，提高城乡低保标准，推进城乡低保一体化。出台农村居民最低生活保障“分类施保”政策。三次启动价调基金为城乡低保对象、优抚对象、农村五保供养对象发放价格补贴3842万元。国家基本药物制度在政府办基层卫生服务机构实现全覆盖，基本药物实现零差率销售。强化旅游惠民，旅游“一卡通”扩展至晋中、阳泉和吕梁，景区由31个增加到44个。全面落实农村新的“五个全覆盖”，农村便民连锁店、行政村体育健身场地、职业高中（中专）学生享受免学费政策提前实现全覆盖，农村街巷硬化开工821个村，完工740个村，竣工5362公里；建成390个农家书屋。这两项今年将实现全覆盖。

社会事业全面加强。全面推进创业就业工程，城镇新增就业人数11.3万人，城镇登记失业率3%。10县（市、区）全部纳入新农保和城镇居民社会养老保险两类国家试点范围，在全省率先实现社会保险政策全覆盖。促进教育公平化进程，全市“联盟校”达到114所。太原大学新校区一期工程建成并投入使用。建立了覆盖城乡的公共文化服务体系。晋剧《傅山进京》荣获“国家十大舞台艺术精品工程”称号，话剧《饭局》荣获国家话剧最高奖“金狮奖”。成功举办第二届太原国际马拉松赛。出色完成第七届全国城市运动会参赛任务。全市人口自然增长率5.14‰。圆满完成第六次人口普查工作。人防、外事、气象、防震、档案、地方志、宗教等工作取得新成绩，慈善、老龄、妇女、关心下一代等事业都有新进步。

社会保持和谐稳定。强化安全生产两个主体责任，持续开展安全生产年活动。煤矿、非煤矿山等11个重点行业连续两年零死亡，各类安全生产事故起数、死亡人数分别下降9.27%、19.23%，没有发生重大、重特大事故，安全生产形势持续稳定好转。加快推进社会管理创新综合试点工作，实施14个重点创新项目，“天网”治安工程、“双百”强基工程和社会组织服务管理三个项目在全国反响良好。全面启动新型警务运行机制，社会治安持续好转。深入开展食品药品安全专项治理和打击制售假冒伪劣商品专项行动。坚持矛盾纠纷排查化解机制和大接访长效机制，全市信访总量下降11.2%。

（六）民主法制建设步伐加快，精神文明创建工作深入开展，城乡群众文明素养进一步提高。

自觉接受人大的法律监督和政协的民主监督，办理省市人大代表建议、政协委员提案800件，办结率100%。支持人大常委会制定、修订6件地方性法规，《太原市价格调节基金管理条例》成为全国第一个规范价调基金的地方性法规。支持政协开展“百名委员视察”活动，对政府职能部门进行民主评议。不断完善基层民主制度，农村两委换届工作基本完成。认真落实党风廉政建设责任制，廉政建设和反腐败斗争取得新成效。扎实推进“法治太原”建设，“六五”普法全面启动。国防后备力量进一步加强，荣获全国双拥模范城“七连冠”。创建全国文明城市取得新成绩，荣获“全国文明城市提名城市”。

（七）第六届中博会综合保障任务圆满完成，招商引资实现新突破，提升了魅力太原新形象。

圆满完成中博会综合保障任务。高标准完成长风文化商务区基础设施、整体配套及我市承担的场馆建设任务。大力推进环境整治，“迎中博、创环境、争一流”活动成效明显。加强文明交通教育，强化运营管理，交通秩序明显改善。各项监管措施坚强有力，应急准备和处置全面及时。氛围营造与形象展示工作效果显著，接待工作细致周到。招商展务成果丰硕，签约项目105个，总投资6617.8亿元，签约项目、总引资额均创历史新高，超过前五届的总和。省委、省政府对我市第六届中博会综合保障工作给予高度评价。

各位代表，我向大会郑重报告，2011年市政府承诺办好的“十件实事”全部完成，投资59.3亿元，为上年的4.3倍。新华社、人民日报、中央电视台等国家级媒体对我市“十件实事”经验做法予以报道，为我省争得了荣誉。我市地区生产总值突破两千亿大关，固定资产投资突破千亿大关，城镇居民收入突破两万元大关，综合实力明显增强，为加快率先转型跨越发展、建设一流省会城市奠定了良好基础。

各位代表，本届政府所取得的成绩，是认真贯彻中央和省各项重大决策的结果，是省委、省政府和市委正确领导的结果，是市人大、市政协和各位代表、各位委员监督支持的结果，是全市人民团结奋斗的结果。在此，我代表市政府，向全市人民，向驻并部队广大指战员、武警官兵和中央、省驻并单位，向各民主党派、工商联、人民团体、无党派人士和所有关心支持太原发展的海内外朋友们，向尽心履职的各位人大代表、政协委员，表示崇高的敬意和衷心的感谢！

回顾本届政府的工作实践，我们深刻体会到：要把太原的各项工作做好，必须强化率先发展意识，坚持科学发展，推进经济发展方式根本转变；必须强化创新意识，努力探索具有太原特色的先行先试之路；必须强化民本意识，切实维护人民群众的根本利益，让人民群众共享改革发展成果；必须强化稳定意识，加强与创新社会管理，维护公平正义，确保省城和谐稳定；必须强化服务意识，优化发展环境，努力营造干事创业的良好氛围。

回顾本届政府的工作实践，我们也清醒地认识到，我市经济社会发展还存在不少困难和问题，主要是：经济发展外部环境严峻复杂，新一轮投资效应尚未充分显现，发展不足仍是当前面临的主要问题；生态环境治理和节能减排形势依然严峻，尤其是冬季大气污染比较突出；经济社会转型进程中各种矛盾凸显，民生改善力度需进一步加大，社会管理加强与创新水平有待进一步提高；干部队伍的素质、能力和作风，与新形势、新任务、新要求还不完全适应，发展环境仍不宽松，影响发展的深层次障碍仍然较多。对此，我们将采取有效措施，切实加以解决。

二、对下届政府工作的建议

新一届政府肩负着推进率先转型跨越发展、圆满完成“十二五”规划、建设一流省会城市的历史重任。今后一个时期，我国仍处于重要战略机遇期，国家大力支持新兴产业发展，加快转变经济发展方式；省委、省政府以推进综改试验区建设为统揽，全力支持太原率先转型跨越发展，我市推进一流省会城市建设面临有利的外部环境。去年以来，我市新上一大批新兴产业项目，投资增速明显加快，产业结构明显优化，发展后劲明显增强。特别是市委、市政府大力创优发展环境，干部队伍的精神面貌和执行力正在发生深刻改变。这些都为我们紧抓发展机遇，做好今后的工作提供了良好条件。只要我们坚定信心，迎难而上，群策群力，就一定能够实现我们的目标。

新一届政府工作的指导思想是：以邓小平理论、“三个代表”重要思想为指导，深入贯彻落实科学发展观，按照省、市第十次党代会部署，以科学发展为主题，以转变经济发展方式为主线，以率先转型跨越为主旋律，以稳中求进为总基调，以建设转型综改试验先导区为龙头，以“四化”建设为重点，以改善民生为出发点和落脚点，加快建设“三个一流”，打牢“一个基础”，强化“四大支撑”，实施对标赶超，全力打造“太原速度”，奋力建设“五区”，把太原建成一流的省会城市，努力成为具有国际影响力的区域性现代化大都市。

新一届政府要全力实现以下奋斗目标：

——综合经济实力显著增强。主要经济指标实现翻番，地区生产总值年均增长13%，固定资产投资年均增长28%，规模以上工业增加值年均增长17%，社会消费品零售总额年均增长15%，财政总收入和一般预算收入年均增长15%。

——转型发展取得明显进展。结构调整取得重大突破，新兴产业体系基本形成，自主创新能力明显提高。万元地区生产总值能耗下降16%，传统产业新型化率达到80%以上，高新技术增加值占地区生产总值的比重达到20%以上。

——城市宜居程度大幅提升。生态环境明显改善，服务功能逐步完善。污染物排放总量下降完成省下达的指标，森林覆盖率力争达到25%，建成区绿化覆盖率达到40%，集中供热覆盖率达到90%以上，良好天气率稳定达到90%以上，公交分担率达到40%。

——人民生活得到较好保障。覆盖城乡的基本公共服务体系日臻完善，社会保持和谐稳定。城镇居民人均可支配收入年均增长13%，农民人均纯收入年均增长13%以上，城镇登记失业率控制在4%以内。

——改革开放步伐不断加快。太原晋中同城化格局基本形成，资源型经济转型综合配套改革先行先试取得显著成效，发展环境显著改善，对外开放广度和深度持续拓展，外贸进出口总额年均增长15%。

围绕上述指导思想和目标，新一届政府要在以下五个重点方面实现突破：

（一）加快工业新型化、服务业和农业现代化，全力建设一流的新兴产业基地。实施工业振兴行动方案，进一步加快发展现代服务业，大力支持都市农业发展。提升改造传统产业，加快发展循环经济，着力提高园区经济发展水平，支持民营经济大发展、大跨越，成为全省高端产业的集聚区。

（二）加快科技进步与人才引进，全力建设一流的自主创新基地。加大高端创新创业人才的引进培养力度，建设一批国家级科技创新平台，攻克一批关键技术，加快成果产业化步伐，形成一批具有核心技术的高科技企业，成为全省科技创新的领先区。

（三）加快市域城镇化和城乡生态化，全力建设一流的现代宜居城市。加快老城改造与古城开发，高标准建设南部区域，完成城中村改造，精细化管理城市。全力推进“气化太原、净化太原、绿化太原”建设，省城环境质量改善“一年初见成效，两年明显改善，三年大见成效，四年目标实现”，打造蓝天白云之城和青山绿水之城。统筹城乡发展，加快新农村建设。努力创建国家环保模范城、国家生态园林城市、国家森林城市，成为全省生态文明建设的示范区。

（四）加快民生改善，加强与创新社会管理，全力打牢社会和谐稳定的基础。加快推进就业、创业、教育、医疗等基本公共服务体系建设，完善社会保障制度，健全社会救助体系，提高保障民生能力。突出抓好稳定物价、食品药品安全、保障性安居工程等工作。全方位推进社会管理创新综合试点工作，坚守安全生产这条红线。扎实创建全国文明城市、全国卫生城市，成为全省和谐社会的首善区。

（五）加快改革步伐和拓展开放领域，全力推进一流发展环境建设。以推进综改试验先导区建设为统揽，深化土地综合利用机制、投融资体制、产业发展机制、文化体制、政府管理体制等各项改革，积极实施一批重大标杆项目，率先走出转型综改发展新路。进一步提高对外开放水平，主动加强与北京对接，加强与中部省会城市的联合协作，加大招商引智的力度，努力成为全省改革开放的先行区。

各位代表，2012年是全面落实省、市第十次党代会部署、完成新一届政府战略任务的首战之年，也是“十二五”规划承上启下的关键之年。政府工作要围绕建设一流省会城市的奋斗目标，加快建设转型综改试验先导区，全面推进一流的新兴产业基地、一流的自主创新基地和一流的现代宜居城市建设，着力保障改善民生，确保社会和谐稳定，以优异的成绩迎接党的十八大胜利召开。

今年全市经济社会发展的主要预期指标是：地区生产总值增长11%；固定资产投资增长30%；规模以上工业增加值增长13%左右；社会消费品零售总额增长16%；地区外贸进出口总额增长14.7%；财政总收入和一般预算收入均增长15%；城镇居民人均可支配收入增长11%；农民人均纯收入增长13%以上；城镇登记失业率控制在4%以内；居民消费价格总水平涨幅控制在4%左右。

约束性指标是：万元地区生产总值能耗下降3.5%；六项污染减排指标、单位地区生产总值二氧化碳排放量和万元工业增加值用水量均完成省下达任务。

新一届政府开局之年的工作重心，就是紧紧围绕稳中求进的总基调，把稳增长、控物价、调结构、惠民生、抓改革、促和谐更好地结合起来，坚持“好”字当头，“快”字领先，坚定不移抓项目，千方百计扩投资，毫不动摇优结构。要积极推进“项目落地年”各项工作，围绕项目建设早谋划、早安排、早投产、早达效。建好用好现有融资平台，支持各开发区融资平台建设，确保重大项目配套资金及时到位。切实破解土地瓶颈制约，加大土地储备力度，积极争取用地指标，挖潜存量建设用地；全面实施土地节约集约利用评价考核机制，转变重大项目报批方式，统筹产业项目向开发区和产业集聚区集中。建立推进重大项目建设的市、县两级领导责任制和联系制，及时研究解决项目建设遇到的问题，保证各类项目特别是重大项目顺利推进和投产达效。

今年着重做好以下八个方面的工作：

（一）加快推进产业结构优化升级，在建设全省高端产业集聚区上取得新突破。

着力推进工业新型化。以园区为载体，以产业链为手段，支持企业向上、向下延伸，推进工业新型化、信息化。制定出台工业振兴行动方案，设立新兴产业发展基金，大力发展新兴产业，切实提高新兴产业占比率。引进三一工业园等50个重大项目，开工建设问天科技云计算中心等30个重大项目，建成晋西春雷高精度铜板带等20个重大项目。巩固煤炭资源整合成果，加快煤炭资源整合保留矿井的现代化矿井建设步伐，支持煤炭企业与电力、焦化、冶金、煤化工、建材等企业的联合，延伸煤炭产业链。重点支持古交新型煤化工循环经济工业园、清徐循环经济园区建设。抓好经济区国家生态工业示范园区、不锈钢园区国家新型工业化产业示范基地建设，培育一批省级循环经济试点企业。

培优做强现代服务业。制定出台加快现代服务业发展的意见，结合工业新型化、市域城镇化的推进，大力发展现代服务业，提升改造传统服务业，加快建设新型服务业产业体系。建设汾东新区综合体、现代物流、旅游会展、文化创意等30个重点传统服务业升级项目和现代服务业项目，带动全市服务业提档升级。优化商业网点规划布局，新建或改造一批农产品批发市场和农贸市场。改造提升消费性服务业，抓好社区菜市场和生鲜超市建设，推进农村社区综合服务中心建设。围绕建设全国优秀旅游目的地城市，加大晋祠片区改造力度，加快建设旅游集散中心和大太原旅游圈，办好第十七届中国北方旅游交易会，旅游总收入突破300亿元。

做精做强现代都市农业。落实中央、省的各项强农惠农富农政策，制定出台支持都市农业发展的意见。夯实农业生产基础，实施一批农田水利工程，推进农业科技工程。按照工业化理念、产业化发展的思路，大力实施“百园兴农”战略，积极发展精品农业、设施农业和休闲观光农业，推进十里汾河都市农业示范区建设。以“一村一品”、“一县一业”为抓手，支持区域特色产业连片发展，提高农业生产的机械化、标准化、规模化、集约化水平。实施农业战略支撑项目10个，打造一批农业科技示范园，农业产业化销售收入力争突破100亿元。切实抓好“菜篮子”工程，实施菜地最低保有量制度，新发展设施蔬菜5000亩，打造蔬菜标准园15个；继续开展农超对接，推进社区便民蔬菜市场建设，畅通农产品运输绿色通道。

提升园区经济带动力。完善园区和县区运行体制，以发展新兴产业和总部经济为重点，大力推进园区经济扩园提质、一区多园。高新区重点推进新区建设，着力打造世界一流的煤化工技术研发集群、电子信息产业集群、新材料和环保节能产业集群。经济区围绕装备制造、新材料、电子信息、食品及农产品加工、生物制药等主导产业发展，重点抓好在建的煤机工业园等重大项目，引进一批新的重大项目。民营区加快推动工业新区和基础区接力发展，打造高端新兴产业集聚区和现代物流服务示范区。不锈钢园区加快不锈钢深加工基地建设，抓好太钢大明等项目建设，全力发展服务于不锈钢产业的现代综合物流中心。清徐开发区重点抓好阳煤化工园区等项目。

推动民营经济大发展。全面落实《大力推进民营经济转型跨越发展的意见》，破解体制性障碍，出台相应配套措施，努力为民营经济创造公平竞争、平等准入的市场环境。建立健全社会化服务体系，搭建民营经济转产转型服务平台，提高民营企业自主创新能力。设立支持民营经济发展专项资金，成立金融业综合协调机构，推动企业上市，扩大直接融资的比例，建设多层次的资本市场，多渠道破解中小企业融资难题。支持兴办实体经济，支持国家微小企业创业园建设，建立成长型中小企业监测机制。将民营经济纳入经济运行调度范围，在煤、电、油、运调度和协调保障中给予同等对待。民营经济增加值占地区生产总值的比重提高1.5个百分点。

加快发展县域经济。以项目和投资为重点，因地制宜、统筹推进县域经济大发展。以“一县一业”为抓手，实现县域经济错位发展、协调发展。加强对县区招商引资工作的考核。加快大县城建设进程，提升城镇建设水平，提高县域统筹协调发展能力。着力推进“简政放权”，做好清徐、古交扩权强县试点建设，增强县域经济自主发展的能力和活力。

（二）加快科技创新和人才强市步伐，在建设全省科技创新领先区上取得新成绩。

提升科技创新能力。开展“产业技术创新工程年”活动，建设一批重点工程中心、企业技术中心、重点实验室，实施科技人员服务企业专项行动和新兴产业“百人千项”专利工程。与中关村合作建设新兴产业科技园。加快“智慧城市”建设步伐。探索科技与金融有效合作新机制，支持高新技术成果产业化，科技计划向新兴产业、优势企业和重点项目“三集中”。全社会研究与开发经费投入增长15%，争取国家各类科技计划项目和经费均增长20%以上；战略性新兴产业领域经省鉴定的科技成果增长30%；专利申请量和授权量均增长30%以上；科技成果转化率达到58%；高技术服务业增加值增长20%；高新技术产业增加值增长20%。

加强人才队伍建设。以打造中西部区域性人才高地为目标，全面加强各类人才队伍建设。以国家“千人计划”和省“百人计划”为重点，完善人才智力引进政策，加强技术研发和项目合作，加快建设院士工作站、博士后工作站。鼓励科技人员带技术带成果服务中小企业。加强与清华大学等国内著名高校合作，培养新兴产业急需的高层次青年科技人才。创新人才开发机制，抓好专业技术人才、高技能人才的选拔培养。

（三）加快推进市域城镇化，在建设现代宜居城市上迈出新步伐。

高水平编制城市规划。围绕建设一流现代宜居城市，启动市区1460平方公里范围内的控规全覆盖工作。高标准深化南部区域特别是汾东商务区和晋阳湖区核心区规划。完善太原—晋中共建区（太原范围）控制性详细规划。做好城郊森林公园总体规划和保税区建设、历史文化名城保护、东西两山绿化规划。围绕公交优先战略，编制完成公共交通专项规划。完成全市绿地系统规划工作。结合全省大水网建设，编制城市水网建设规划。

高标准建设基础设施。重点实施美术馆等中博会后续完善工程5项；新兰路改造等城市道桥新建改建工程18项；汾河治理美化工程1项；启动轨道交通工程。实施86条小街巷的综合改造工程。汾东商务区重点建设中央景观带及两侧路网等6类项目；晋阳湖区加快建设晋阳湖周边重点地段路网等工程；南站片区年内完成西广场工程主体，力争成为新的亮点片区。加快明太原县城保护开发建设。启动双塔景区（含朝阳片区）建设。启动“公交都市”建设。建设快速化公交专用道路网，开工建设客运东南站和9个公交场站，实施西山旅游及防火通道等15项工程。抓好西南环征地拆迁等7项铁路建设工程。高标准抓好供水、供气、供热、环卫、污水处理、垃圾处理等市政公用基础设施建设。加强电网建设，建设坚强智能电网。

提升精细化管理水平。以“五城联创”为抓手，深入推进“城乡清洁工程”，继续抓好创建达标活动，重点打造一批精品片区。围绕市政公用设施安全稳定运行，推进城乡管理物联网项目建设，逐步实现对公共设施的全方位、全天候预警监控。加强“数字城管”、“法治城管”、“服务城管”、“和谐城管”建设，着力解决群众反映的热点和难点问题。严格土地管理，强化执法监察，严守耕地红线。完善城市建设、运行监管体系，实现全市土地统一收储，提高土地集约化利用水平。

推进小城镇和新农村建设。加大百镇建设力度，积极争取国家和省小城镇建设专项补助资金，集中力量打造2到3个示范镇。结合“一村一品”建设，扎实推进新农村建设和扶贫工作，切实抓好60个一流新农村和集中连片示范区建设。全面实现农村新的“五个全覆盖”，提高农村公共服务能力。

（四）加快推进城乡生态化，在建设全省生态文明示范区上打造新亮点。

全面改善省城环境质量。狠抓集中供热全覆盖、气化太原、城中村整村拆迁改造、污染企业搬迁、水污染治理等五大工程建设。年内集中供热扩网1500万平方米，力争2000万平方米。完成古交—太原等三项气源引入工程，对河西地区21万户煤气用户进行天然气置换。建设一批加气站，解决加气难问题。推进常年运行燃煤锅炉替代工程。新增28个城中村改造项目。启动第一热电厂搬迁。全面开展汾河边山支流截污整治工作。加快城市污水处理设施及配套管网建设。市区地表水环境功能区水质达标率稳定在60%以上。严格遏制扬尘污染，强化机动车尾气监管。开展细微颗粒（PM2.5）监测，市区良好天气率达到82.2%以上，市区空气质量达到国家二级标准，省城环境质量改善初见成效。

做好节能减排和资源综合利用工作。全面开展百家重点企业“节能环保对标”行动，以300兆瓦以上燃煤发电机组脱硝为重点，大力推进污染减排。继续实行能源消费总量控制，严格执行固定资产投资项目节能评估与审查制度。大力推广节能、可再生能源等先进技术，做好以节电为重点的强制性能源审计工作。对新建建筑推行65%节能标准，把长风商务区建成绿色建筑示范区。强化节约集约用地考核，推进浅层地温能调查评价和开发利用试点工作。

加大生态建设力度。完善生态建设投融资机制，吸引更多市场主体参与绿化，完成造林48.5万亩，其中，西山城郊森林公园完成6万亩。推广实施树木直补政策，做好林改配套试点工作和林地利用保护规划。继续“绿染龙城、花满并州”三年植绿大行动，实施21项园林绿化重点工程，建成区绿化覆盖率、绿地率均提高1个百分点，人均公园绿地增加0.5平方米。提高8万农村人口饮水标准。综合治理水土流失面积20万亩。实施“两河一湖”水系连通治理工程。加强汾河水库环境整治，集中式饮用水源地水质达标率稳定保持100%。

各位代表，推进城乡生态化，建设生态文明，是全市人民的热切期盼，是关乎长远、惠及子孙的大事。我们要下大决心、使大力气，把改善空气质量特别是整治冬季大气污染作为民生的头等大事来抓，改造城中村，停用小锅炉，拔掉黑烟囱，搬走污染源，治理臭水河，让并州大地天更蓝、地更绿、水更清、空气更清新、环境更宜人！

（五）加快民生改善和发展社会事业，在建设全省和谐社会首善区上再上新台阶。

全面推进民生与社会事业发展。推进创业型城市建设，实现创业带动就业的倍增效应，重点解决高校毕业生、困难群体、农民工等群体的就业问题，全市城镇新增就业9.5万人，就业困难群体就业1.06万人。加强民生公共服务设施建设，提高社会综合保障服务水平。“百校兴学”的重心从校舍建设向改善教育教学设施转变。新增100所省义务教育标准化学校。推进太原大学“专升本”工作。实施“百院兴医”工程，改善医疗卫生条件。加强重要商品特别是生活必需品的产运销衔接，支持粮食、蔬菜流通体系建设。提升公众防震减灾意识，提高城乡防灾减灾综合能力，推进人防工程建设。对全市社区健身设施进行更新改造。积极开展慈善救助活动。启动市档案馆新馆、市老年活动中心项目。稳定低生育水平，提高人口素质，推进“幸福家庭”创建工作。在全省率先推行峰谷电价。其它各项社会事业要强化“对标”，争创一流，取得新成绩，实现新进步。

加强和创新社会管理。继续做好社会管理创新试点工作。加强流动人口管理，实施居住证管理制度，实现均等化服务。严厉打击“两抢一盗”、非法集资等犯罪行为。开展平安创建活动，推进“天网”治安工程，强化社区警务，全面构建新型社会治安防控体系。加大对城乡结合部、城中村等重点区域的整治力度。加强社区建设，推进“双百”强基工程。加强房地产市场监管，严厉打击违法售房行为，坚决制止违法建设、违法占地。加快建立“源头预防、过程控制、应急处置”三道防线，积极化解信访案件，妥善处置信访问题。完善价格调控政策和应对市场价格异动的预案，严肃查处各类价格违法行为。充分发动群众，加强机制建设，拓宽群众监督渠道，形成铲除制假售假、私挖乱采等各种违法犯罪活动的强大社会力量。

毫不动摇抓好安全生产。深入开展对煤矿、非煤矿山、危险化学品等重点行业的“打非治违”行动。严格落实安全生产企业主体责任和政府属地监管责任，推进企业安全生产标准化建设，建立并推行安全生产测评体系。加大对校车、火灾隐患等交通、消防安全问题的整治力度。健全食品药品安全监管体系，提高食品药品安全保障水平，严厉打击假冒伪劣食品药品犯罪。建设市食品药品综合监督检测中心。集中力量开展农产品、畜产品、食品安全整顿，建立生产与流通全过程质量监控机制，严厉查处滥用激素、添加剂等行为，打造“放心餐桌”。健全完善各项应急预案，提高突发事件应急处置能力。

各位代表，保障和改善民生、建设和谐太原，是人民政府义不容辞的神圣责任。我们要以高度负责的精神，将民生发展之路越走越宽，越走越顺，让全市人民生活的更加幸福、更有尊严。

（六）加快推进综改试验先行先试，在建设全省改革开放先行区上打开新局面。

着力推进综改试验先导区建设。加快武宿综合保税区筹建工作，力争7月封关运行。抓好以项目促转型工作，实施太重高速列车轮轴及齿轮箱国产化、东西山绿化等重大标杆项目。推动试点板块建设，抓好尖草坪区、万柏林区和古交市转型综改试点工作。推进国家循环经济标准化试点工作和太榆科技创新城建设。创新矿业用地管理机制，实施城乡建设用地增减挂钩试点规划方案。发挥企业转型主体作用，推进国有企业兼并重组，发挥国有资本放大效应。发行地方建设债券，深化城市投融资等体制机制改革。推进“企业一套表”联网直报统计改革。

提高对外开放水平。加强与京津地区的对接和环渤海城市的合作交流，把太原建设成北京最大的副中心、卫星城和产业承接地。积极推进太原都市圈建设，以太原晋中共建区建设为抓手，统筹推进城市规划、土地利用、产业布局、基础设施建设和生态保护，实现优势互补、经济互动、资源共享。坚定不移实施开放带动战略，拓展国际交流合作，加大以企引企、以商引商、以侨引商力度，特别是加强工业项目招商，引进一批符合发展规划的大项目、好项目，实际使用外资增长15%。抓好第六届中博会签约项目的审批、履约与跟踪服务，提高项目履约率和资金到位率。推动外贸增长方式转变，扩大机电和高新技术产品出口份额。拓展对外经济合作领域，重点在服务外包和技术引进上下功夫。

深化公共财政改革。认真落实积极的财政政策，加大对转型跨越发展和综改区先行先试的财政支持力度。深化预算管理制度改革，健全预算执行管理制度，强化预算绩效管理。提高财政收入质量，确保财政增收的稳定性与可持续性。继续优化财政支出结构，向改善民生倾斜，提高财政保障水平。

（七）加快推进文化大发展大繁荣，在建设文化强市上实现新超越。

全面落实《关于深化文化体制改革加快建设文化强市的实施意见》，以高度的文化自觉和文化自信推动文化大发展大繁荣。

加强精神文明建设。实施铸魂聚力工程，推进社会主义核心价值体系建设，宣传实践“三个核心价值观”。引深公民道德建设工程，开展社会志愿服务活动，建设与“一流省会城市”相适应的思想道德体系。完善创建全国文明城市工作机制，做好全国城市文明程度指数测评工作。巩固群众性精神文明创建活动成果，深化文明村镇、文明家庭等创建活动，引导各类新经济组织、社会组织、基层组织积极参与创建活动。

加快发展文化产业。实施文化产业振兴工程，精心打造太原晋商文化产业园区，建设以高新区为中心的文化创意产业集聚区和数字出版基地，加快发展动漫游戏产业。抓好长风商务区会展、演出、科技、旅游一体化建设。做好文化产业项目招商引资工作，组织参加第七届中国北京国际文化创意产业博览会和第六届西部文化产业博览会。

继续繁荣文化事业。深化文化体制改革，健全文化管理体制和生产经营体制。做好第四批国家级非物质文化遗产保护项目的申报和认定工作，加快推进晋阳古城国家考古遗址公园等项目建设。实施精品塑造工程，推出更多文艺精品。实施强基惠民工程，完善公共文化服务体系，完成乡镇文化站建设，县级文化馆、图书馆全部达到国家标准，推行公共图书馆、文化馆的免费开放；建设209个农家书屋和121个村级文化活动场所，实现全覆盖。开展“文化太原、幸福龙城”群众性广场文化活动。办好新年音乐会，打造城市文化新名片。

各位代表，太原是具有五千年文明史、二千五百多年建城史的国家历史文化名城，我们要像抓经济建设一样抓文化建设，努力当好建设文化强省的排头兵，为建设一流省会城市提供强大的精神动力和良好的文化环境。

（八）加快民主法制建设，在构筑转型跨越发展政治保证上谱写新篇章。

推进民主政治建设。认真执行人大及其常委会的决议、决定，自觉接受人大、政协和社会各界的监督，广泛听取各民主党派、工商联、无党派代表人士的意见和建议。积极支持人民政协履行政治协商、民主监督、参政议政职能，提高人大代表建议和政协提案的办理质量和落实效率。完善基层民主自治和企事业单位民主管理制度。保障和支持监察、审计部门依法独立履行监督职责。

加快依法治市进程。健全地方立法机制，加快制定和完善结构调整、城乡统筹、循环经济、社会管理等方面的地方性法规。加强法治政府建设，提高行政执法的水平和能力。推进司法体制改革，提升公正司法的水平。继续加强地方治理、行业治理、基层治理，完善依法治理结构。认真实施“六五”普法，加快“法治太原”建设。支持国防和军队建设，巩固“双拥模范城”创建成果。

三、切实加强政府自身建设

各位代表，新一届政府肩负着光荣而艰巨的历史使命。我们必须下大力气抓好政府自身建设，尽职尽责，真抓实干，砥砺前行，以优异的成绩不负党和人民的重托。

（一）服务发展大局，提高履职能力。各级领导干部要牢固树立一切为了太原发展的全局观，提高发展太原、服务人民的能力，成为做好工作的行家里手，成为抓好工作落实的高手。要以求真务实的实干精神和实际行动，勇敢担当起我市在全省示范发展、引领发展、带动发展的神圣使命，干出经得起历史检验的业绩。

（二）加快职能转变，提高行政效能。坚持依法科学民主决策，规范行政决策程序，完善行政决策风险评估机制，加强重大决策跟踪反馈和责任追究。按照公开透明、便民高效要求，深入开展政务环境“对标”活动；按照“两集中、两到位”的要求，全面实施新的固定资产投资项目联合审批流程，落实“一站式”办公、限时办结等制度；加快电子政务建设，推进政府信息公开，创新绩效考核办法，全方位优化政务环境，打造全省最好、全国一流的发展环境。

（三）着力改进作风，提高执行能力。保持奋力赶超、争先争上的精神状态，发扬只争朝夕、坚韧不拔的创业作风，坚决治理慵懒散现象。把“项目化”作为推进工作的重要载体，各项工作都要做到目标明确、措施具体、检查到位、落实有力。加强调查研究，深入基层、深入群众，开展领导干部下乡住村包村增收活动，使政府工作、政策措施更加符合民意。加强“三位一体”政务督查，提高执行力和工作效率。强化行政问责，严肃查处有令不行、有禁不止和不作为、慢作为、乱作为等行为。

（四）加强廉政建设，做到清正廉洁。全面落实党风廉政建设责任制，扎实推进惩治和预防腐败体系建设。大力纠正部门和行业不正之风，切实解决损害群众利益的问题。厉行勤俭节约，推进公务接待、公车使用、因公出国三项制度改革，加强对财政资金、政府重大投资项目等的审计监察，从源头上治理腐败。严肃查处各类违法违纪行为，营造风正气顺、廉洁高效的发展环境。

各位代表，新一届政府既要抓好转型跨越这件大事，也要抓好自身建设这件要事，更要抓好民生保障这件实事。今年市政府要着力办好以下十件实事：一是把改善环境质量作为最大的民生工程，完成17个城中村整体拆除，对21个城中村实施集中供热改造，关停分散燃煤采暖小锅炉508台，拔掉黑烟囱1.81万根；对154台常年运行燃煤锅炉实施清洁能源替代；8月底前对太化、煤气化等8家重点污染企业实施关停；完成玉门河、九院沙河、南沙河、北沙河4条河流全河段、汾河西干渠上兰至晋阳湖段和杨兴河截污工程。二是进一步解决困难群众住房难问题，开工建设保障性住房5万套。三是加快“公交都市”建设，进一步解决群众出行难问题，新增公交车600台，建设公交专用道90公里；建设智能化公交系统，迎泽大街、南内环街、新建南路公交站台建成乘客诱导系统；筹建公共自行车租赁系统，投入1.2万辆自行车；开通太原到阳曲、清徐的公交车。四是方便群众就医，启动“百院兴医”一期工程，实施25项医疗卫生机构改扩建项目。五是继续实施“百校兴学”工程，新建、改扩建50所项目学校、30所公办幼儿园。六是关爱残疾人，为2500名贫困残疾人提供康复救助，为社区（村）、康复机构配发135套残疾人康复训练器材；为260户农村贫困残疾人家庭实施危房改造。七是关爱老人，为全市孤寡、独居、空巢老人配发爱心一键通1万部；在100个社区开展居家养老服务，为60岁以上的独居、空巢、困难家庭老人发放居家养老服务券。八是进一步解决群众如厕难问题，新建公厕100座，改造60座。九是开展文化惠民活动，为全市1000个农村文化大院、农家书屋配备文化专管员；文化精品惠民演出500场。十是开展科技惠民活动，在100个蔬菜生产基地、大型超市、菜市场和学校食堂示范推广蔬菜安全检测系统。

各位代表，新的形势催人奋进，新的征程任重道远。让我们紧密团结在以胡锦涛同志为总书记的党中央周围，在省委、省政府和市委的坚强领导下，在市人大、市政协的监督支持下，紧紧依靠全市人民，坚定信心，扎实工作，为我市率先转型跨越发展、建设一流的省会城市和具有国际影响力的区域性现代化大都市而努力奋斗！
